# Editorial: Investigating the role of periodontal microbiota in health and disease

**DOI:** 10.3389/froh.2025.1682460

**Published:** 2025-08-21

**Authors:** Thuy Do, Divyashri Baraniya, Nezar Al-Hebshi

**Affiliations:** ^1^ Division of Oral Biology, School of Dentistry, University of Leeds, Leeds, United Kingdom; ^2^ Oral Microbiome Research Laboratory, Maurice H. Kornberg School of Dentistry, Temple University, Philadelphia, PA, United States

**Keywords:** periodontitis, microbiome & dysbiosis, systemic health, systemic disease, oral biofilm

**Editorial on the Research Topic**
Investigating the role of periodontal microbiota in health and disease

Being home to over 700 microbial species, the oral cavity harbours the second most complex and diverse microbial ecosystem in the human body that plays an indispensable role in maintaining both localized oral health and broader systemic well-being. Disruptions to the delicate balance of this microbial community, termed dysbiosis, are now recognised as central to the pathogenesis of highly prevalent oral diseases, most notably periodontitis. Beyond the direct impact on oral tissues, accumulating evidence also underscores the profound systemic implications of oral dysbiosis, including obesity, diabetes, cardiovascular disease, Alzheimer's, colorectal cancer, non-alcoholic fatty liver disease, inflammatory bowel disease, and rheumatoid arthritis (1, 2).

This collection of five papers advances the understanding of the features of oral microbial dysbiosis associated with oral and systemic diseases, how it can be shaped by demographic and socio-economic factors and its diagnostic potential. The papers are varied in focus, ranging from exploring the oral microbiome in specific periodontitis genotypes, to reviewing its role in infective endocarditis, and finally to assessing the prevalence of oral parasites in vulnerable populations. They don't only delve into fundamental microbial mechanisms, such as dysbiosis, the roles of keystone pathogens, virulence factors, and community shifts, but also directly explore their clinical applicability. This includes validation of diagnostic indices like the subgingival microbial dysbiosis index (SMDI) (Molli et al. 2023), the assessment of the diagnostic potential of specific bacteria like *Filifactor alocis* (Faisal et al. 2025), implications for antibiotic prophylaxis (Falconer et al. 2024) and the critical need for improved patient education (Selahbarzin et al. 2024). This dual focus on basic science and clinical translation within a single research topic is indicative of a field actively striving to bridge the gap between discovery and patient care.

Molli et al. investigated the subgingival bacteriome in Moroccan adolescents with Grade C periodontitis (GCP)- previously called aggressive periodontitis- associated with the *A. actinomycetemcomitans*-JP2 genotype. They found a significantly altered microbial composition in GCP cases, with enrichment of known periodontal pathogens (*Porphyromonas gingivalis*, *Tannerella forsythia*, *Treponema denticola*, *Fretibacterium spp., etc.)* in addition to non-conventional taxa like *Pseudomonas oral taxon C61* and *Enterobacter cloacae*. The previously described SMDI (3) effectively discriminated GCP with 95% accuracy The study concluded that bacteriome dysbiosis might be a prerequisite for the activity of the JP2 genotype,. Indeed, a key finding revealed that JP2-positive subjects within the control group clustered with JP2-negative healthy samples, rather than with GCP cases. This intriguing observation suggests that the mere presence of the JP2 genotype might not be sufficient to trigger disease, instead, a pre-existing state of bacteriome dysbiosis could be a prerequisite for the full expression of JP2 genotype activity or its heightened pathogenicity. This refines the keystone pathogen hypothesis, where a highly virulent keystone pathogen may not be sufficient to initiate or drive disease, but rather, an altered microbial environment is required to enable the pathogen's virulence to manifest and orchestrate destructive immune responses. This understanding shifts the focus of therapeutic and preventive strategies from solely targeting specific keystone pathogens to understanding and modulating the host-microbiome interactions that create a permissive or susceptible environment for their activity. It opens avenues for therapies aimed at restoring microbial homeostasis or disrupting the dysbiotic environment, rather than just pathogen eradication.

In a different geographical context, Kabbashi et al. conducted a pilot study to characterize subgingival bacterial communities in periodontitis patients from the Western Cape, South Africa. They revealed distinct bacterial communities between periodontitis cases and controls, with cases dominated by *Fusobacterium*, *Porphyromonas*, and *Treponema*, while controls were rich in *Streptococcus*. The study also observed shifts in dominant genera across periodontitis grades (e.g., *Fusobacterium* more abundant in Grade C), suggesting population-specific microbial profiles and supporting the polymicrobial synergy and dysbiosis model. The finding of distinct compositional shifts in dominant genera between Grade B and Grade C periodontitis (e.g., *Fusobacterium* dominance in Grade C, *Porphyromonas* and *Treponema* in Grade B) is highly significant in that specific composition and relative abundance of certain taxa might serve as a microbial signature for the *rate* of disease progression. This implies that different microbial communities might drive distinct pathogenic trajectories, rather than simply contributing to overall disease severity. This opens a promising new avenue for research into developing microbial biomarkers specifically for predicting periodontitis *progression rate* (grading).

Further insight into the microbiome associated with periodontitis is provided by Faisal et al. who assessed the prevalence and diagnostic potential of *Filifactor alocis* in subgingival biofilm samples from periodontitis stage 3 and 4 patients in Iraq. They found *F. alocis* to be highly prevalent (78.9%) and its load significantly increased with disease severity (stage 4 vs. stage 3) and increasing probing pocket depth (PPD) and clinical attachment loss (CAL). The study concluded that *F. alocis* possesses significant diagnostic potential to differentiate between periodontitis stages, particularly in deep periodontal pockets.

Selahbarzin et al. evaluated the prevalence of oral parasites, *Entamoeba gingivalis* and *Trichomonas tenax*, in children with intellectual disability (ID) in Lorestan province, Iran. They reported a significantly higher prevalence of these parasites in ID children (40.5% and 42.8% respectively by microscopic and PCR) than in healthy controls. Key risk factors identified included inadequate tooth brushing, urban residence, lower parental education, and lower family income, underscoring the need for enhanced public and oral health strategies for this vulnerable population. The explicit identification of socioeconomic factors (parental education, family income, urban residence) and fundamental oral hygiene practices (inadequate tooth brushing) as independent risk factors for the prevalence of oral parasites in children with ID directly reinforces the understanding that systemic health outcomes originating from oral conditions are not solely biological phenomena but are deeply and inextricably intertwined with broader social determinants of health. Public health interventions aimed at improving oral health and, by extension, mitigating systemic health risks, must adopt a multi-pronged approach. This approach needs to transcend individual clinical care by actively addressing socioeconomic disparities and promoting basic, accessible oral hygiene education and resources, particularly within underserved and vulnerable communities. This shifts the paradigm from a purely medical problem to a significant public health equity challenge.

Falconer et al. provided a narrative review exploring the substantial links between oral health and infective endocarditis (IE). The paper highlighted that the oral microbiome is deeply implicated in IE pathogenesis, with bacteraemia commonly occurring from daily activities and dental procedures. It identified *Staphylococcus aureus* and oral streptococci (e.g., *S. sanguinis*) as key causative agents, emphasised the role of biofilm formation in hindering treatment, and discussed diagnostic techniques and the need for improved patient awareness and collaborative pathways between cardiology and dental teams.

To advance the field of oral health, several critical knowledge gaps must be addressed. Future research should move beyond mere taxonomic profiling to understand the functional contributions, metabolic activities, and virulence mechanisms of identified microbial communities. This necessitates the application of multi-omics approaches, including metagenomics, metatranscriptomics, and metabolomics. Rigorous longitudinal studies are essential to establish definitive causal relationships between specific microbial shifts, host responses, and disease outcomes, rather than relying solely on cross-sectional associations.

The research presented in this topic collectively highlights significant advancements in understanding the oral microbiome's intricate role in health and disease. These studies reinforce that microbial dysbiosis is a central driver of periodontitis, with a growing recognition of both traditional and emerging, non-conventional pathogens. Crucially, the profound and multifaceted connections between oral health and systemic conditions, particularly infective endocarditis, are further elucidated, emphasizing the oral cavity as a critical gateway to systemic health. The demonstrated diagnostic potential of microbial biomarkers for periodontitis staging, alongside the validation of dysbiosis indices and the use of advanced sequencing technologies, heralds a new era of precision diagnostics in oral health. Looking forward, the insights gleaned from these papers underscore the imperative for continued, interdisciplinary research, focusing on functional microbial analyses, longitudinal studies, and the development of personalised, population-specific interventions. Ultimately, fostering greater public awareness and strengthening collaborative efforts across medical and dental disciplines will be paramount to transforming oral and systemic health outcomes globally.
